# Prognosis of patients with fulminant myocarditis managed by peripheral venoarterial extracorporeal membranous oxygenation support: a retrospective single-center study

**DOI:** 10.1186/s40560-014-0069-9

**Published:** 2015-02-08

**Authors:** Tomohiro Nakamura, Kohki Ishida, Yousuke Taniguchi, Tomu Nakagawa, Masaru Seguchi, Hiroshi Wada, Yoshitaka Sugawara, Hiroshi Funayama, Takeshi Mitsuhashi, Shin-ichi Momomura

**Affiliations:** Department of Medicine, Jichi Medical University Saitama Medical Center, Saitama, Japan; Cardiovascular Division, Jichi Medical University Saitama Medical Center, 1-847 Amanuma, Omiya-ku, Saitama Japan

**Keywords:** Fulminant myocarditis, Extracorporeal membranous oxygenation support, Prognosis

## Abstract

**Background:**

Peripheral venoarterial extracorporeal membranous oxygenation (ECMO) support is effective in patients with cardiogenic shock or fatal arrhythmia due to fulminant myocarditis. The clinical courses of fulminant myocarditis are still uncertain; therefore, it is difficult to determine the appropriate time for discontinuing ECMO or converting to a ventricular assist device. The purpose of this study was to investigate the prognosis of patients with fulminant myocarditis managed by ECMO.

**Methods:**

Twenty-two consecutive patients with fulminant myocarditis managed by peripheral venoarterial ECMO between 1999 and 2013 were enrolled.

**Results:**

Survival to discharge was 59% (13 patients), and in-hospital mortality was 41% (9 patients). The age in the survivor group was significantly lower than that in the non-survivor group (survivor group vs. non-survivor group; 36.5 ± 4.1 vs. 60.2 ± 5.0 years, *p* = 0.001). Although the ECMO support duration was similar between the groups (181 ± 22 vs. 177 ± 31 h), the rate of complication related to ECMO was significantly lower in the survivor group (15.3% vs. 66.6%, *p* = 0.02). When comparing the laboratory data during ECMO management between the groups, the serum bilirubin level on day 7 was significantly lower in the survivor group (total: 4.6 ± 2.8 vs. 13.7 ± 10.8 mg/dL, *p* = 0.014; direct: 2.2 ± 0.5 vs. 9.8 ± 4.5 mg/dL, *p* = 0.009).

**Conclusions:**

Fulminant myocarditis is associated with high mortality rates despite ECMO. An older age and complications related to ECMO are associated with poor prognosis.

## Background

Fulminant myocarditis, which is defined as inflammation of the myocardium, is a rapidly progressive life-threatening disease accompanied by cardiogenic shock and heart failure [1,2]. When conventional therapy cannot support the circulatory condition, mechanical circulatory support is required. Previous studies showed that mechanical circulatory support is effective in patients with cardiogenic shock due to fulminant myocarditis, and it was possible to achieve an overall survival rate of 50%–70% [3-7]. Peripheral venoarterial extracorporeal membranous oxygenation (ECMO) support can be introduced quickly and easily without the need for a cardiovascular surgeon compared to a ventricular assist device (VAD), and we applied peripheral venoarterial ECMO as a first line of mechanical circulatory support. However, we have to consider converting to a VAD if cardiac function cannot be recovered even with ECMO management. The aims of the present study were to investigate the prognosis of patients with fulminant myocarditis supported by ECMO and to clarify the limitations of ECMO.

## Methods

### Patient population

In a retrospective chart review from 1999–2013, we identified 22 consecutive patients with fulminant myocarditis managed by peripheral venoarterial ECMO at Jichi Medical University Saitama Medical Center. The diagnosis of myocarditis was based on the following clinical findings: 1) a recent medical history consistent with flu-like symptoms such as respiratory or gastrointestinal symptoms; 2) ST-T abnormalities detected by electrocardiography; 3) significant changes in echocardiographic features; 4) positive findings of inflammation (e.g., a fever of >38°C, increased white blood cell count, and increased C-reactive protein level); 5) absence of critical stenosis observed via coronary angiography; 6) sudden onset of cardiogenic shock; and 7) requiring ECMO for prolonged cardiogenic shock. The pathological etiology of fulminant myocarditis was not included in these criteria. The induction criteria for ECMO were cardiogenic shock refractory to inotropic agents, intra-aortic balloon pumping (IABP), or life-threatening arrhythmia.

### The device and management

The peripheral venoarterial ECMO system consists of a centrifugal pump, a polypropylene hollow-fiber membrane oxygenator, and a heparin-coated circuit (Terumo Inc., Tokyo, Japan). We used a 15-French (Fr) arterial cannula with a length of 15 cm and a 21-Fr venous cannula with a length of 50 cm, both of which were inserted via the femoral vessels by using the Seldinger technique. Mechanical circulation was established with venous blood drainage from the right atrium and arterial blood return to the iliac artery.

The management and weaning of ECMO were performed according to the previously described guideline [7]. Low-dose heparin was continuously administrated to maintain an activated clotting time of 150–200 s. To prevent poor venous drainage, right atrium pressure was maintained (>10 mm Hg) with adequate fluid administration, and blood transfusion was performed to maintain a hemoglobin level of >10 g/dL and a platelet level of >50,000/μL. A distal perfusion catheter into the superficial femoral artery or dorsal pedis artery was required to prevent distal leg ischemia.

The initial flow rate was 3.0–3.5 L/min to assist recovery from peripheral circulatory failure. In all patients, ECMO was introduced in the catheter laboratory, and the initial flow rate and rotation speed of ECMO were appropriate. According to the indicators of peripheral circulatory failure (e.g., arterial blood gas analysis, mixed venous oxygen saturation, lactic acid, and urinary output), the flow rate of ECMO was decreased. When the flow rate reached 1.0 L/min, the patients could be weaned off ECMO if their vital signs and the abovementioned indicators were acceptable.

### Data collection and statistical analysis

Blood samples were obtained every 4 h until the peak creatinine kinase (CK) and CK-MB levels were determined, and thereafter, they were collected every 24 h until the patients recovered or died. We have investigated the ECMO-related complications including leg ischemia, stroke, major bleeding, and multiple organ failure (MOF). The definition of major bleeding was the uncontrolled hemorrhage requiring the additional transfusion at vascular access sites, retroperitoneal space, or gastrointestinal tract.

Data were expressed as the frequency and percentage for categorical variables and the mean ± standard deviation for continuous variables. We compared data between the survivors and non-survivors. Continuous data were analyzed by using the Student *t*-test or the Mann–Whitney *U*-test. Categorical data were analyzed by using the Fisher exact test. For all analyses, a two-tailed *P* value of <0.05 was considered statistically significant. All statistical analyses were performed by using the SPSS statistical software, version 13.0 for Windows (SPSS, Chicago, IL, USA). All subjects enrolled in this research have given their informed consent which has been approved by my institutional committee on human research, and this protocol has been found acceptable by them.

## Results

Between 1999 and 2013, we included 22 patients with fulminant myocarditis who were managed with ECMO. Thirteen patients (59%) were discharged from the hospital. One of these 13 patients underwent heart transplantation after converting to the left VAD, and she was discharged in excellent condition. Table 1 shows the patients’ baseline clinical characteristics. On admission, the circulatory condition and laboratory data were similar between the survivor group and the non-survivor group. The average age of those in the survivor group was significantly lower than that in the non-survivor group (36.5 ± 4.1 vs. 60.2 ± 5.0 years, *p* = 0.001).

**Table 1:** **Baseline characteristics of patients with fulminant myocarditis with extracorporeal membranous oxygenation (ECMO) support**

	**Survivors ** **(** ***n*** **= 13)**	**Non-survivors ** **(** ***n*** **= 9)**	***P*** **value**
Age, years	36.5 ± 14.7	60.2 ± 14.9	0.001
Male sex	6 (46%)	4 (44%)	0.64
Systolic BP, mm Hg	84.5 ± 21.7	81.4 ± 27.3	0.78
Heart rate, bpm	110.0 ± 33.3	92.0 ± 26.1	0.22
Presence of pulseless VT or Vf	9 (69%)	6 (67%)	0.90
LVDd, mm	46.0 ± 7.7	44.0 ± 5.4	0.62
LVDs, mm	40.7 ± 7.3	36.4 ± 4.2	0.25
LVEF, %	22.9 ± 8.5	23.6 ± 12.6	0.88
Laboratory data on admission			
WBC (/μL)	10,213.9 ± 5,431.6	11,937.8 ± 9,399.0	0.59
GOT (IU/L)	768.6 ± 864.1	1,599.8 ± 2,759.7	0.40
GPT (IU/L)	540.6 ± 726.9	1,044.2 ± 1,882.0	0.46
LDH (IU/L)	2,187.9 ± 2,198.0	2,815.1 ± 4,245.1	0.65
Total-Bil (mg/dL)	0.72 ± 0.29	0.85 ± 0.49	0.46
Direct-Bil (mg/dL)	0.28 ± 0.11	0.37 ± 0.31	0.36
BUN (mg/dL)	26.7 ± 12.4	38.9 ± 29.0	0.26
Creatinine (mg/dL)	1.47 ± 0.76	1.48 ± 0.98	0.99
Max. CK (IU/L)	3,090.2 ± 4,416.1	3,148.0 ± 4,456.2	0.98
Max. CK-MB (IU/L)	154.9 ± 279.8	113.2 ± 70.1	0.67

Data are presented as the mean ± standard deviation or as the number of patients (%).

*BP* blood pressure, *VT* ventricular tachycardia, *Vf* ventricular fibrillation, *LVDd* left ventricular end-diastolic dimension, *LVDs* left ventricular end-systolic dimension, *LVEF* left ventricular ejection fraction, *WBC* white blood cell, *GOT* glutamic oxaloacetic transaminase, *GPT* glutamic pyruvic transaminase, *LDH* lactate dehydrogenase, *Bil* bilirubin, *BUN* blood urea nitrogen, *Max*. maximum, *CK* creatine kinase.

Table 2 compares the treatment and ECMO management between the groups. The use of steroid, IABP, and continuous hemodiafiltration was similar between the groups. Although the ECMO duration was similar between the groups (survivor group vs. non-survivor group; 181 ± 22 vs. 177 ± 31 h, respectively; *p* = 0.15), the rate of complications related to ECMO was significantly lower in the survivor group (15.3% vs. 66.6%, *p* = 0.02). Complications such as major bleeding and MOF were more common in the non-survivor group.

**Table 2:** **The treatment and management of ECMO**

	**Survivors ** **(** ***n*** **= 13)**	**Non-survivors ** **(** ***n*** **= 9)**	***P*** **value**
Steroid therapy	7 (54%)	2 (22%)	0.15
IABP use	13 (100%)	8 (89%)	0.41
CHDF use	2 (15%)	3 (33%)	0.32
Total operating time of ECMO, h	181 ± 22	177 ± 31	0.15
Complications associated with ECMO			
Total	3 (23%)	8 (89%)	0.004
Leg ischemia	0 (0%)	2 (22%)	0.16
Stroke	0 (0%)	2 (22%)	0.16
Bleeding	2 (15%)	6 (67%)	0.02
Multiple organ failure	2 (15%)	6 (67%)	0.02

Data are presented as the mean ± standard deviation or as the number of patients (%).

*IABP* intra-aortic balloon pump, *CHDF* continuous hemodiafiltration.

**Figure 1:**
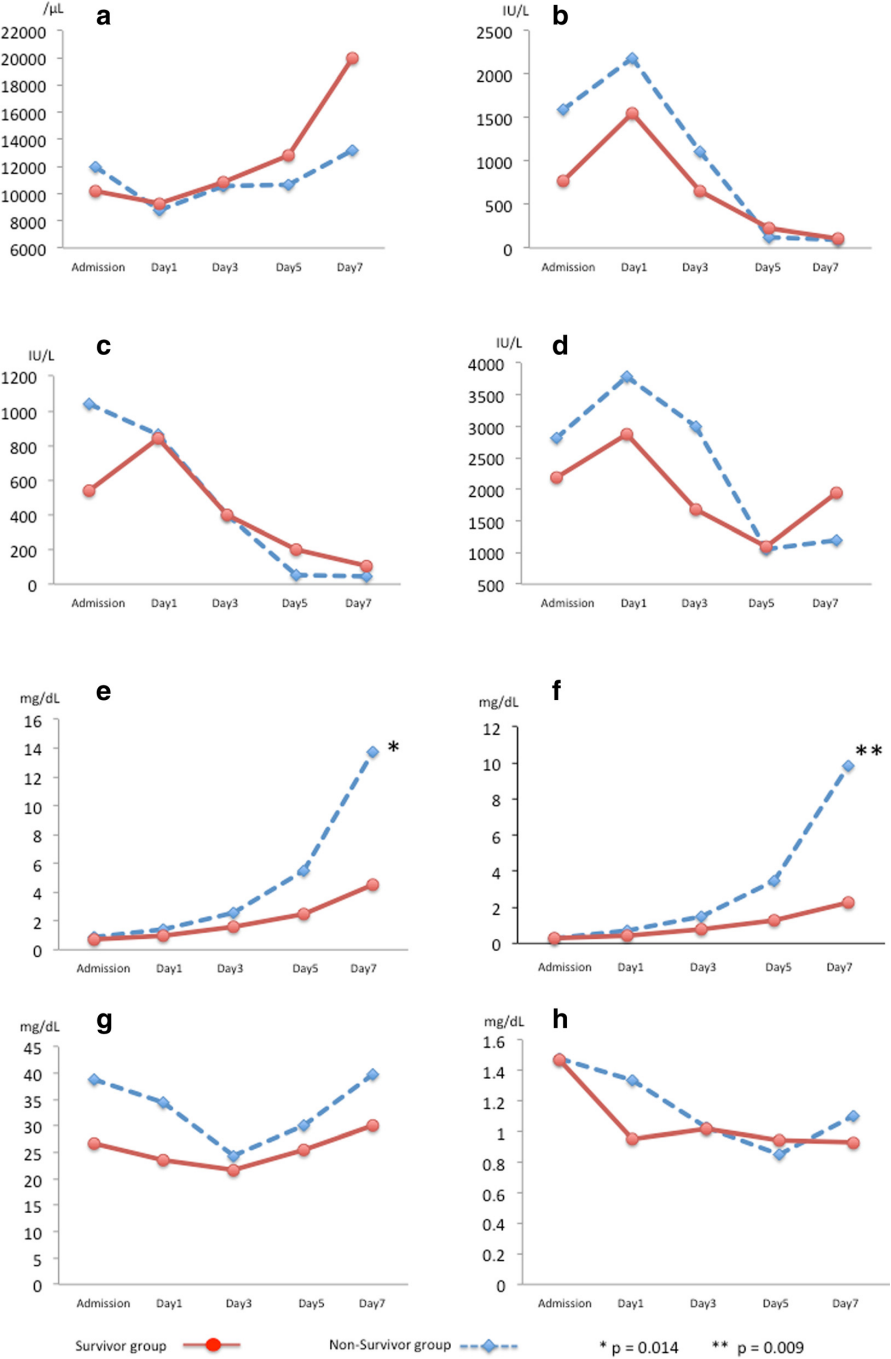
**Serial changes of markers during extracorporeal membranous oxygenation support. (a)** White blood cell (WBC), **(b)** glutamic oxaloacetic transaminase (GOT), **(c)** glutamic pyruvic transaminase (GPT), **(d)** lactate dehydrogenase (LDH), **(e)** total bilirubin, **(f)** direct bilirubin, **(g)** blood urea nitrogen (BUN), and **(h)** creatinine. There were no significant differences between the survivor group and non-survivor group, except for in the serum bilirubin levels. The serum total and direct bilirubin levels on day 7 in the survivor group were significantly lower compared with those on day 7 in the non-survivor group (total bilirubin: 4.6 ± 2.8 vs. 13.7 ± 10.8 mg/dL, *p* = 0.014; direct bilirubin: 2.2 ± 0.5 vs. 9.8 ± 4.5 mg/dL, *p* = 0.009).

Figure 1 shows the serial changes in the laboratory data, including the white blood cell count and levels of glutamic oxaloacetic transaminase, glutamic pyruvic transaminase, lactate dehydrogenase, total bilirubin, direct bilirubin, blood urea nitrogen, and creatinine, during ECMO management. The serum levels of total bilirubin and direct bilirubin on day 7 were significantly lower in the survivor group (total bilirubin: 4.6 ± 2.8 vs. 13.7 ± 10.8 mg/dL, *p* = 0.014; direct bilirubin: 2.2 ± 0.5 vs. 9.8 ± 4.5 mg/dL, *p* = 0.009).

## Discussion

Fulminant myocarditis, which is characterized by extensive inflammatory infiltration and numerous foci of myocyte necrosis, is a rapidly progressive life-threatening disease, and survival rates have been reported as 50%–70% [1-7]. Our study found that the survival rate was 59% in patients with fulminant myocarditis managed by peripheral venoarterial ECMO. And elderly patients who might be in vulnerable conditions were associated with poor prognosis. Although the baseline characteristics except for age were similar between the survivor group and the non-survivor group, it was expected that the reserve of end-organ function is low in elderly patients. Considering that we do not treat pediatric patients with fulminant myocarditis and we excluded adult patients who did not require ECMO, our findings are acceptable.

Cardiopulmonary support systems play an important role in patients who develop cardiogenic shock or fatal arrhythmia. Pages et al. [8] reported that peripheral venoarterial ECMO was as efficient as bi-VAD in patients with fulminant myocarditis related to cardiogenic shock, because it facilitated renal and hepatic recovery. Acker et al. [4] suggested that the survival rates in cases by using peripheral venoarterial ECMO were higher than in cases by using VAD. When compared to VAD, peripheral venoarterial ECMO could be introduced quickly and easily to prevent hemodynamic deterioration. In Japan, a therapeutic guideline by Aoyama et al. [7] recommended that ECMO should be instituted if the patient with fulminant myocarditis does not recover from circulatory failure despite the use of cardiotonic or vasopressor drugs and IABP treatment. However, continued use of ECMO causes many problems, including bleeding, hemolysis, leg ischemia, or MOF, and ECMO is not suitable for long-term support. Previous studies investigating patients with acute coronary syndrome requiring ECMO reported that the complications related to ECMO were associated with poor prognosis [9,10]. A study in patients with fulminant myocarditis by Aoyama et al., as previously described, reported that leg ischemia and MOF related to ECMO were poor prognostic factors [7]. Our study findings were similar considering the incidence rate of ECMO-related complications; in particular, bleeding events and MOF were significantly greater in the non-survivor group. This suggests that we should pay careful attention to prevent complications related to ECMO in an effort to improve the prognosis of fulminant myocarditis.

MOF is one of the serious and important problems that can occur during ECMO management. Liver failure is especially difficult to treat, even though cardiopulmonary failure and renal failure can be supported by VAD and hemodialysis. Peek et al. [11] reported that a high bilirubin level in patients managed by ECMO was associated with high mortality. Unosawa et al. [12] suggested that ECMO should be converted to VAD before the total bilirubin level increases to 3.0 mg/dL. Imamura et al. [13] reported that bilirubin or creatinine levels adjusted by age can be predictors of reversibility of end-organ dysfunction in patients with advanced stage heart failure. In our study, the serum bilirubin levels in the non-survivor group significantly increased during ECMO management, which might reflect the progression of MOF. According to these findings, the serum bilirubin level might help us to manage the patients with fulminant myocarditis during ECMO. Therefore, we have to consider this when converting peripheral venoarterial ECMO to VAD in patients whose cardiac functions are not improved and in those whose serum bilirubin levels have suddenly increased after ECMO was introduced.

This study has several limitations. First, it was a retrospective study at a single center, and the number of subjects is small. Second, we excluded pediatric patients, and the minimum age in our study was 14 years old. Thus, it is unclear whether our conclusions can be applied to pediatric patients. Third, since the study period was 1999–2014, improvement in ECMO management may affect the prognoses. Fourth, in our hospital, only one patient in the survivor group was converted from peripheral venoarterial ECMO to VAD because of fulminant myocarditis; therefore, we have to admit a lack of experience in using VAD for treating fulminant myocarditis. Thus, the prognosis after conversion to VAD is unknown.

## Conclusions

In conclusion, the mortality of fulminant myocarditis with hemodynamic collapse is still high even under ECMO management. We should pay careful attention to elderly patients and the complications related to ECMO, which are both associated with poor prognosis.

## Abbreviations

ECMO: Extracorporeal membranous oxygenation support
VAD: Ventricular assist device
IABP: Intra-aortic balloon pumping
CK: Creatinine kinase
MOF: Multiple organ failure
